# 加速康复外科：肺癌手术日间化现状与策略

**DOI:** 10.3779/j.issn.1009-3419.2020.01.01

**Published:** 2020-01-20

**Authors:** 国卫 车

**Affiliations:** 610041 成都，四川大学华西医院胸外科 Department of Thoracic Surgery, West China Hospital, Sichuan University, Chengdu 610041, China

**Keywords:** 加速康复外科, 日间手术, 分级诊疗, 肺肿瘤, Enhanced recovery after surgery, Day surgery, Graded diagnosis and treatment, Lung neoplasms

## Abstract

加速康复外科（enhanced recovery after surgery, ERAS）理论、手术器械和治疗病种的变化，均需要重新审视现在的临床治疗观念和操作流程。ERAS理念从兴起到完善，为复杂却风险低的手术日间化提供了理论和技术支持。结合最近的国内外临床实践，以肺癌为例，分析肺癌手术日间化面临的问题与应对策略。从以下几方面论述：一是肺癌患者由住院手术转为日间手术（ambulatory surgery day surgery）的必要性与可行性；二是肺癌手术日间化的团队及平台建设；三是肺癌日间手术操作流程及围手术期管理需要优化；四是利用“分级诊疗-日间手术”模式保障患者安全；五是肺癌日间手术临床应用前景。

## Abstract (en)

It is time to review the current clinical treatment concepts and operational procedures that the changes of enhanced recovery after surgery (ERAS) theory, surgical instruments and disease treatment types. It is support of theoretical and technical of ERAS concept from rise to perfection for day surgery with complex but low risk operation. Combined with the recent clinical practice at home and abroad, this study review the problems and measures of day surgery in patients with lung cancer. First, the necessity and feasibility of patients with lung cancer undergoing day surgery instead of inpatient surgery. Second, establishment of day surgery team and platform in lung cancer surgery. Third, operational procedures and perioperative management of day surgery need to be optimized. Fourth, the "graded diagnosis and treatment-day surgery" model was used to ensure patient safety. Fifth, Clinical application prospect of ambulatory surgery for lung cancer.

外科学在本世纪的进步体现在微创技术和加速康复外科（enhanced recovery after surgery, ERAS）理念的有机融合，二者共同促进外科手术向更小创伤和更低风险发展^[1]^。外科技术、设备和理念的更新，需要我们重新审视外科治疗的临床观念和操作流程，尤其是否可以使部分外科住院手术日间化管理？外科手术日间化，不但加快病床周转、提高医疗资源使用效率、减少院内感染等优点^[2]^，更有助于探索适应现代外科技术和理念的围手术期管理流程。减少医疗干预，增加医疗服务，提高患者就医满意度。又可探索使患者在家庭医生，社区医院，专科医院之间合理流动（分级诊疗），并得到优质服务的途径。胸外科目前开展的日间手术，倾向于简单的手术，肺癌手术日间化需要我们在以下几方面进行思考与探索：一是肺癌手术日间化的必要性及可能性；二是肺癌手术日间化的团队及平台建设；三是围手术期流程如何优化；四是如何利用现有的分级诊疗体系保障病人安全。

## 1 肺癌患者由住院手术转为日间手术的的必要性与可行性

日间手术（day surgery）是指在24 h内完成患者入院、手术治疗、术后观察和康复出院的一种外科治疗模式，其概念1909年由英国小儿外科医生James Nicoll提出，2003年国际日间手术协会将其定义。2012年中国日间手术合作联盟（China Ambulatory Surgery Aliance, CASA），结合国际经验和国内实际医疗情况，将日间手术重新定义为“患者24 h内完成手术或操作并出院^[3]^。目前我国整体的医疗规范及医疗同质化水平、分级诊疗制度与国际先进国家和地区还存在一定差距，在现有医疗环境下发展日间手术，必需在保障医疗安全及质量的前提下，才能最大程度地追求日间手术管理模式下要求的患者24 h内出院的高效率治疗模式。其初衷不能否认是为部分中、小手术患者提供了一种新型医疗服务，由于同时涉及手术、麻醉及围术期管理等多个环节，日间手术的开展在我国仍处于起步阶段。

部分肺癌手术是否有转为日间手术的必要性和可行性如何呢？从以下几方面看必要性：一是由当前中国医疗国情，医疗资源分布不均衡、分级诊疗制剂不健全，导致患者仍习惯于挤大医院、找名专家，形成了“看病难、看病贵”的怪圈。在当前情况下，大医院或专科医院只有通过加速患者周转，提高效率，是日间手术开展的国情所在；二是肺癌的疾病谱也在发生变化，体现在人们对健康体检的重视和低剂量螺旋计算机断层扫描（computed tomography, CT）临床应用，使年轻肺癌患者越来越多，相对于老年患者（合并疾病多），其围手术期风险相对降低；从患者（免去患者去挤大医院，又能得到团队合理、便捷治疗）和社会（使患者尽快身心都回归正常生活和社会）角度看，都需要我们开展并推行部分肺癌患者手术日间化。肺癌手术日间化有可行性吗？首先是早期肺癌患者增多，手术复杂性相对降低，麻醉及术中风险也较少；其次是加速康复理念及微创技术的充分应用使患者术后快速康复，使术后恢复在社区和家庭成为可能。再次麻醉技术、术中操作、疼痛控制技术、管理管理和护理流程的优化，极大的减少了医疗干预，使患者术后康复加快，为患者围手术期的快速康复和医疗安全提供了保障；最后各家医院的日间手术中心所拥有的信息技术、随访机制也为患者术后社区或家庭康复提供了应急处理、便捷、安全途径^[4]^。目前状况下，有必要在我国部分医疗中心开展日间肺癌手术，且从技术及操作上是可行的。

## 2 肺癌手术日间化的团队及平台建设

结合国内的实际医疗情况，中国日间手术合作联盟将日间手术重新定义为“患者24 h内完成手术或操作并出院；并强调：一是日间手术是对患者有计划进行除门诊手术外的手术和操作；二是由于特殊病情变化及患者自身病情转归需要延长住院时间的患者，最长时间不超过48 h^[5]^。目前我国大陆整体的医疗规范及水平与国际先进国家和地区还存在一定差距，在现有医疗环境下发展日间手术，应有医疗团队及日间手术中心平台作为保障医疗安全及质量的前提。

肺癌手术日间化的平台（管理模式）及团队如何建设呢？我国日间手术的管理模式分为：集中、分散、集中与分散并行^[3]^。“集中管理”指是建立专业的综合管理病区，以集中收住院、安排手术和术后护理及随访的一体化管理模式。四川大学华西医院日间手术采取的是集中管理模式，而日间肺癌手术更接近集中分散管理相结合模式，从而最大限度发挥各学科最大优势。既日间手术中心负责术前准备、麻醉评估、围手术期管理和术后随访由它们统一完成；胸外科同时派出医生进行全流程参与，并制订肺癌患者日间手术标准、手术方法、管道操作方案、申请伦理及医生准入标准等。团队如何组建呢？总结肺癌住院手术患者日间化管理经验，需要麻醉科、日间手术室、疼痛科、营养科、康复科、日间手术中心医生及护理和胸外科医生组成。具体分工如下：胸外科医生评估患者是否能够进行日间手术（目前主要依据年龄和肿块大小进行判定），并完成术前检查及术前准备、手术操作及术后管理。麻醉科需在门诊麻醉评估、并制订麻醉方法及注意事项；疼痛科需在门诊根据病人情况，制订合理的围手术期及术后疼痛管理方案，目前主要采用术中肋间神经阻滞方法（切口上下3个肋间），不用镇痛泵；术后辅以静脉推注甾体类镇痛药（如凯纷和特耐），必要时口服甾体类镇痛药^[6]^。营养科对患者的饮食进行专业化管理，例如术前给患者喝糖水，术后2 h会为患者准备开胃汤，助于患者胃肠功能快速恢复，改善头晕的症状，并且为患者定制当晚及第二天早上餐食，给予出院后饮食指导^[7]^。康复科负责术前心肺功能评估、术后运动及咳嗽管理^[8]^；出院后居家康复。日间手术中心医护团队负责围术期管理并根据病情协调各个科室及时处理紧急情况。肺癌手术日间化成功实施需多学科团队协作，离不开“医、护、麻”等相关科室的全力合作。“虚拟团队在日间手术整个围手术期发挥了重要作用，既分工又协作，不仅提高医疗质量并保障了患者安全；同时，方便患者就医，减少医疗费用。

## 3 肺癌日间手术操作流程及围手术期管理需要优化

肺癌日间手术总的处理原则是“减少医疗干预，增加医疗服务”。肺癌日间手术和住院手术目前均以微创手术为主，但日间手术更加着重围手术期的术前精准评估、术中合理优化、术后按需服务。主要从以下几方面着手：一是术中强调尽量缩短麻醉和手术时间，术前麻醉评估气管插管的难度，并进行呼吸道的准备（如戒烟、肺康复训练等）；二是术中手术器械的优化，缩短非手术而增加的手术时间（如器械准备不到位、清点器械时间、不必要器械的安装与拆卸等）^[9, 10]^，手术方法也以缩短手术时间作为主要考虑方面（当前主要是3孔法单向式胸腔镜肺叶切除术）。三是减少各种管道的应用：不使用尿管，为了避免术后发生危险，术前建议患者把尿液排空。护士也会随时关注病膀胱情况，如果出现尿潴留马上采取热敷、诱导处理，并尽量使患者自行排尿。胸腔引流管目前采用18号硅胶双腔尿管，优势是管腔透明，管径粗细可以满足引流气体和液体，且因管腔内囊腔可注水，而不需要切口处用缝线固定，可减轻疼痛及不影响切口愈合^[11, 12]^。三是控制麻醉液体，合理使用抗生素。四是做好镇痛工作，尽量减少阿片类药物应用，优先选择使用副反应小且效果好的药物^[11]^。五是减少不必要的监护：按需提供服务，按需调整医疗干预，按需给患者检查。为了监测患者生命体征，通常医生都会给术后患者身上安插各种监测设备、电极片等，还有各种管道。“我们不把患者‘捆’在床上”。优化服务，让护士每隔2 h对患者的血压和饱和度进行测量，为给予患者更多方便和自由。

## 4 利用“分级诊疗-日间手术”模式保障患者安全

日间手术短平快的模式特点，出院后延续服务是保障医疗质量和实现患者快速康复的重要举措。四川大学华西医院日间手术中心以护理为主导开展日间手术出院后延伸服务^[4]^。主要有以下举措：一是术前宣教、沟通，通过术前沟通，说明日间手术的特点及注意事项，公众号普及一些术后问题的处理知识，减少患者内心的顾虑。二是建立追踪随访制度，出院后由专人对术后患者进行电话随访、QQ、微信、互联网信息平台在线沟通，时刻关注患者术后的康复情况，而且患友之间能够相互交流和鼓励。三是与基层医疗机构建立了系统的转诊合作，实现患者随访信息无缝对接。四是利用远程会诊平台，实现患者手术在医院，康复在基层；外地患者可就近到社区医院进行诊治，避免劳途奔波。

利用华西医院日间手术中心现有的平台优势，结合肺癌日间手术的特点，我们对现在的流程进行了优化和补充，主要有以下几方面：一是术前检查及宣教，胸外科手术医生与日间手术中心护士一起，消除患者对手术的恐惧和担心。二是手术团队医生全程参与术后管理，若有意外或紧急发生，马上得到及时处理，需要时马上转住院病房处理。三是出院后建立专科医生日间医护、当地医院、患者及时联系的网络（目前主要微信群及随访平台）平台。

## 5 肺癌日间手术临床应用前景

肺癌日间手术的开展，不仅方便了患者就医、节约了患者费用，而且能够让患者迅速回归社会和生活，有利于患者的身心健康。同时肺癌日间手术的开始，标志着加快康复外科理念的真正实现。减少医疗干预，增加医疗服务，让患者真正体会到个体化与人性化的医疗，提高患者就医满意度，才是日间手术临床应用的真正意义所在。肺癌日间手术的实现，能够促进患者能够在家庭医生、社区医生、专科医生之间合理地流动，改善医疗服务，发挥各级医疗资源的合理配用，实现患者、医院、社会的双赢局面

肺癌日间手术的患者，总费用比住院手术至少要降低20%。患者的就医感受度和满意度均提高，患者对日间手术的满意度达到了95%以上^[5]^。使医院紧张的医疗资源能够最大化，能够更好地为更多患者提供优质的服务，真正实现了患者利益最大化。胸科肺癌手术成功日间化，是“医、护、麻”等相关的科室全力合作的结果，真正做到了以临床上以常见问题为导向，以患者为中心，多方共同解决患者的问题，提高医疗质量、护理的成效。多学科团队协作，分级诊疗-日间手术模式的推进，必将引领肺癌日间手术的更好、更快发展！
